# Microbial biotechnology: from synthetic biology to synthetic ecology

**DOI:** 10.1007/s44307-024-00054-4

**Published:** 2025-01-03

**Authors:** Qingyun Yan, Yuzhen Ming, Jianzhong Liu, Huaqun Yin, Qiang He, Juan Li, Mingtao Huang, Zhili He

**Affiliations:** 1 Marine Synthetic Ecology Research Center, Southern Marine Science and Engineering Guangdong Laboratory (Zhuhai), Zhuhai, 519082 China; 2 School of Life Sciences, Sun Yat-Sen University, Guangzhou, 510275 China; 3 School of Minerals Processing and Bioengineering, Central South University, Changsha, 410006 China; 4 Department of Civil and Environmental Engineering, The University of Tennessee, Knoxville, TN 37996 USA; 5 College of Agronomy, Hunan Agricultural University, Changsha, 410128 China; 6 School of Food Science and Engineering, South China University of Technology, Guangzhou, 510641 China

**Keywords:** Microbial biotechnology, Synthetic biology, Synthetic ecology

Microorganisms are ubiquitous and almost omnipotent on Earth. Over the past few decades, microbial biotechnology has revolutionized fields ranging from medicine to environmental remediation by harnessing the power of microorganisms for diverse applications. More recently, the emergence of synthetic biology and synthetic ecology has introduced new tools and strategies, enabling us to engineer and manipulate microbial systems for beneficial outcomes.

This special issue under the topic of *Microbial Biotechnology* compiles the latest advancements in microbial studies, with a particular focus on using crucial microbial technologies (e.g., genomics, metagenomics, synthetic biology, synthetic community construction) to foster our understanding of key microorganisms in both natural and man-made ecosystems. We have now published research papers that highlight the microbially-driven nutrient cycles in water (Li et al. 2024a), soil (Li et al. 2024b), sediment (Zhang et al. 2024a) and air (Zhang et al. 2024b) environments, as well as review/research articles on synthetic biology (Xu et al. 2024; Zhou et al. 2024; Zhuang et al. 2024).

Synthetic biology involves the design and construction of novel biological systems or redesign of existing ones for specific applications (Endy 2005). This field has enabled genetic engineering of microorganisms (Fig. 1) to produce biofuels, pharmaceuticals, and industrial chemicals more efficiently and sustainably (Keasling 2012). Advances in genome editing tools, such as CRISPR-Cas9, have facilitated precise manipulation of microbial genes and modification of metabolic pathways and genetic circuits (Doudna & Charpentier 2014). These engineered microorganisms offer sustainable alternatives to traditional chemical synthesis methods and hold great promises for addressing global challenges in energy, health, and food production. Synthetic biology also provides new opportunities for environmental remediation, offering possibilities of engineered microorganisms as novel catalysts to degrade pollutants, detoxify contaminants, and restore ecosystems (Lovley 2003). However, many of these applications target pure cultures, which only account for a small fraction of the microbial world. Furthermore, the behavior of single microorganisms may differ significantly from that in microbial communities due to complex interactions among different constituents of the community. Such limitations may hinder the applications of synthetic biology in natural ecosystems.

**Fig. 1:**
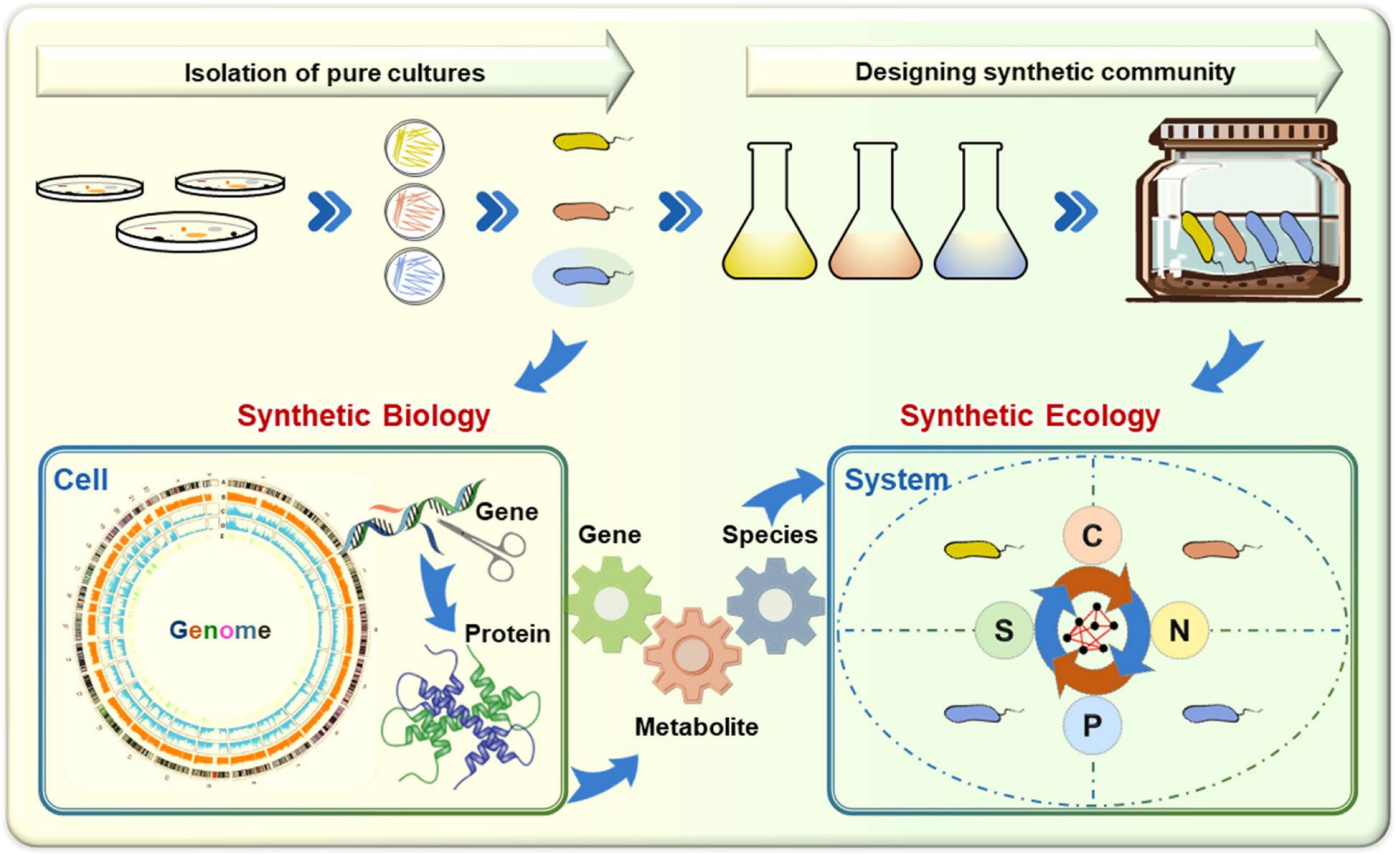
A conceptual model of microbial biotechnology from synthetic biology to synthetic ecology. C-carbon, N-nitrogen, P-phosphorus, S-sulfur

Synthetic ecology aims to understand biotic and abiotic interactions and applications of de novo ecological communities towards a desirable purpose through design, construction, optimization and analysis of single organisms (Fig. 1). Synthetic ecology can offer a more streamlined model to investigate fundamental ecological principles and construct communities with desired functions. Moreover, synthetic communities could exhibit better performance and be more tractable to control than natural communities. Few studies have shown that synthetic microbial communities (SynComs) were tested in microbiome-host interactions (Carlström et al. 2019; Fu et al. 2020), crop resiliency (Carrión et al. 2019), and human gut disease treatment (van der Lelie et al. 2021). However, many of these applications have emerged from empirical correlations rather than mechanistic understandings, often relying on trial-and-error, highlighting the ongoing challenges for designing SynComs based on first principles.

Recent advances in microbial cultivation, synthetic biology, metabolic engineering, and computational biology have paved the way for more effective construction of SynComs. An emerging strategy is to design the metabolic interactions among community members to strengthen their interactions. Various mathematical modelling approaches are available to characterize biological and metabolic characteristics within a microbial community (Zaramela et al. 2021). For example, computational approaches can more precisely identify critical pathways that influence microbial interactions (Jing et al. 2024). Combining multi-omics and computational approaches further allows to establish stable syntrophic interactions with optimized growth (Zuñiga et al. 2020). Integration of techniques in synthetic biology and metabolic engineering can further facilitate the design of synthetic communities to achieve functional specialization, such as identifying highly conserved promoter regions to enhance gene expression or knocking out specific genes to force cells to obtain nutrients from other community members.

Albeit these advances, synthetic ecology is still in its infancy. Selecting desirable candidate microorganisms for the synthetic community construction remains a huge challenge. A key issue to maintain the stability and functionality of the synthetic microbial systems over time and across diverse environmental conditions (Brune & Bayer 2012). Also, elucidating the interactions between community members is required but poorly understood, challenging our capability to rationally design synthetic microbial communities. In addition, constituents of the synthetic ecosystem may also exhibit unpredictable behaviors, genetic instability, or ecological interactions that could compromise their designed performance in practical applications (Khalil & Collins 2010). Nevertheless, synthetic ecology is a shortcut for exploring fundamental principles and realizing biotechnological potentials. This insight will enhance our understanding of microbial ecology and facilitate its applications in the medicine, agriculture, industry and environment.

The Marine Synthetic Ecology Research Center (MSEC) was established in 2024 at the Southern Marine Science and Engineering Guangdong Laboratory (Zhuhai). MSEC’s primary mission is to design and construct synthetic communities to elucidate the mechanisms of biological interactions and their relationships with the environment. Effective synthetic communities will be developed to utilize marine biological resources, strengthen ecological services, protect marine environments, and mitigate global changes through microbiome engineering.

## References

[CR1] Brune KD, Bayer TS. Engineering microbial consortia to enhance biomining and bioremediation. Front Microbiol. 2012;3:203. 22679443 10.3389/fmicb.2012.00203 PMC3367458

[CR2] Carlström CI, Field CM, Bortfeld-Miller M, Müller B, Sunagawa S, Vorholt JA. Synthetic microbiota reveal priority effects and keystone strains in the Arabidopsis phyllosphere. Nat Ecol Evol. 2019;3(10):1445–54. 31558832 10.1038/s41559-019-0994-z PMC6774761

[CR3] Carrión VJ, Perez-Jaramillo J, Cordovez V, Tracanna V, De Hollander M, Ruiz-Buck D, Mendes LW, van Ijcken WF, Gomez-Exposito R, Elsayed SS. Pathogen-induced activation of disease-suppressive functions in the endophytic root microbiome. Science. 2019;366(6465):606–12. 31672892 10.1126/science.aaw9285

[CR4] Doudna JA, Charpentier E. The new frontier of genome engineering with CRISPR-Cas9. Science. 2014;346(6213):1258096. 25430774 10.1126/science.1258096

[CR5] Endy D. Foundations for engineering biology. Nature. 2005;438(7067):449–53. 16306983 10.1038/nature04342

[CR6] Fu H, Uchimiya M, Gore J, Moran MA. Ecological drivers of bacterial community assembly in synthetic phycospheres. Proc Natl Acad Sci USA. 2020;117(7):3656–62. 32015111 10.1073/pnas.1917265117 PMC7035482

[CR7] Jing J, Garbeva P, Raaijmakers JM, Medema MH. Strategies for tailoring functional microbial synthetic communities. ISME J. 2024;18(1):wrae049. 38537571 10.1093/ismejo/wrae049 PMC11008692

[CR8] Keasling JD. Synthetic biology and the development of tools for metabolic engineering. Metab Eng. 2012;14(3):189–95. 22314049 10.1016/j.ymben.2012.01.004

[CR9] Khalil AS, Collins JJ. Synthetic biology: applications come of age. Nat Rev Genet. 2010;11(5):367–79. 20395970 10.1038/nrg2775 PMC2896386

[CR10] Li XT, Deng XS, Hou DW, Zeng SZ, Deng ZX, Zhou RJ, Zhang LY, Hou QL, Chen Q, Weng SP, He JG, Huang ZJ. Effects of water ammonia nitrogen on hemolymph and intestinal microbiota of *Litopenaeus vannamei*. Adv Biotechnol. 2024a;2:1. 10.1007/s44307-023-00008-2 PMC11740837 39883200

[CR11] Li P, Tian YH, Yang K, Tian MJ, Zhu Y, Chen XY, Hu RW, Qin T, Liu YJ, Peng SG, Yi ZX, Liu ZX, Ao HJ, Li J. Mechanism of microbial action of the inoculated nitrogen-fixing bacterium for growth promotion and yield enhancement in rice (Oryza sativa L.). Adv Biotechnol. 2024b;2:32. 10.1007/s44307-024-00038-4 PMC11709144 39883349

[CR12] Lovley DR. Cleaning up with genomics: applying molecular biology to bioremediation. Nat Rev Microbiol. 2003;1(1):35–44. 15040178 10.1038/nrmicro731

[CR13] van der Lelie D, Oka A, Taghavi S, Umeno J, Fan T, Merrell KE, Watson SD, Ouellette L, Liu B, Awoniyi M. Rationally designed bacterial consortia to treat chronic immune-mediated colitis and restore intestinal homeostasis. Nat Commun. 2021;12(1):3105. 34050144 10.1038/s41467-021-23460-x PMC8163890

[CR14] Xu P, Lin NQ, Zhang ZQ, Liu JZ. Strategies to increase the robustness of microbial cell factories. Adv Biotechnol. 2024;2:9. 10.1007/s44307-024-00018-8 PMC11740849 39883204

[CR15] Zaramela LS, Moyne O, Kumar M, Zuniga C, Tibocha-Bonilla JD, Zengler K. The sum is greater than the parts: exploiting microbial communities to achieve complex functions. Curr Opin Biotechnol. 2021;67:149–57. 33561703 10.1016/j.copbio.2021.01.013

[CR16] Zhang DD, Yu H, Yu XL, Yang YC, Wang C, Wu K, Niu MY, He JG, He ZL, Yan QY. Mechanisms underlying the interactions and adaptability of nitrogen removal microorganisms in freshwater sediments. Adv Biotechnol. 2024a;2:21. 10.1007/s44307-024-00028-6 PMC11740870 39883300

[CR17] Zhang HT, Hu J, Peng X, Zhou L, Zhang T, Zhang YF, Yin HQ, Meng DL. Impacts of ammoniacal odour removal bioagent on air bacterial community. Adv Biotechnol. 2024b;2:8. 10.1007/s44307-024-00016-w PMC11740863 39883329

[CR18] Zhou XL, Zhang MS, Zheng XR, Zhang ZQ, Liu JZ. Increasing the robustness of *Escherichia coli* for aromatic chemicals production through transcription factor engineering. Adv Biotechnol. 2024;2:15. 10.1007/s44307-024-00023-x PMC11740835 39883341

[CR19] Zhuang ZK, Wan GY, Lu XC, Xie LH, Yu T, Tang HT. Metabolic engineering for single-cell protein production from renewable feedstocks and its applications. Adv Biotechnol. 2024;2:35. 10.1007/s44307-024-00042-8 PMC11709146 39883267

[CR20] Zuñiga C, Li T, Guarnieri MT, Jenkins JP, Li C, Bingol K, Kim Y, Betenbaugh MJ, Zengler K. Synthetic microbial communities of heterotrophs and phototrophs facilitate sustainable growth. Nat Commun. 2020;11(1):3803. 32732991 10.1038/s41467-020-17612-8 PMC7393147

